# Editorial: Women in the history, culture and sociology of sports: 2021

**DOI:** 10.3389/fspor.2022.1027702

**Published:** 2022-09-08

**Authors:** Lucie Schoch, Sheryl Clark, Jessica Francombe-Webb

**Affiliations:** ^1^Institute of Sport Sciences, Université de Lausanne, Lausanne, Switzerland; ^2^Department of Educational Studies, Goldsmiths University of London, London, United Kingdom; ^3^Department for Health, University of Bath, Bath, United Kingdom

**Keywords:** women, gender, sport, history, sociology, culture

Gender inequalities in academia are persistent and present global challenges. The literature on “gender and science” underlines how careers in science and academia are still subject to discrimination according to gender. This is made visible by the number of women academics: at present, <30% of researchers worldwide are women. Furthermore, women remain particularly underrepresented at higher professorial levels and leadership positions (1). Among the different cumulative social mechanisms explaining this “leaky pipeline” within the academic track—such as university being a gendered organization, with masculine norms predominating in the scientific workplace (2)— is the conflict of working life/family life (3). This latter point has been visible during the early months of the COVID-19 pandemic. Given that most academics were forced to work from home, the competing demands for familial duties have penalized the scientific productivity of women, while, on the opposite side, male researchers have increased their scientific production (4).

These gender biases are particularly noticeable in the field of sport sciences (5). Women are underrepresented at policy level, as well as in conference committees, keynote speaker lists, panels and other events. They remain markedly underrepresented in leading authorship and editorial board positions in sport sciences, despite small increases (6). Sport and exercise science remain “a man's game” (7).

In order to change traditional mindsets, gender equality must be promoted, stereotypes defeated, and girls and women should be equally represented in the proportion of (sport) researchers worldwide. Therefore, this volume is a collection of papers aimed at promoting the work of women researchers, across all fields of sports science broadly defined. Each paper within this edited collection has been led by a researcher who identifies as a woman. The papers provide advances in theory and methodology, with commitments to praxis and change.

“*How Heavy Lifting Lightens Our Lives: Content Analysis of Perceived Outcomes of Masters Weightlifting*” analyzes self-reported effects of participating in Masters-level Olympic weightlifting on other aspects of life (Huebner et al.). The study design allows authors to have a systematic look at gender and age range differences. Results show that weightlifting has a positive impact on physical health (strength, mobility, fitness) and on psychological (mental health benefits, stress reduction) and social aspects such as community connections. Female lifters mentioned psychological benefits such as increased confidence and help with stress and depression more commonly than male lifters; older lifters were more likely than middle-aged lifters to mention physical health benefits. The study highlights the benefits to groups who were previously marginalized within this sport, in particular older women weightlifters who are countering both aging and gender stereotypes.

“*Accessibility, Agency, and Trust: A Study About Equestrian's (Online) Learning Repertoires*” focuses on the increase in online sources of information and their consumption, within the context of equestrian sport (Broms et al.). This context is important because the production/consumption of horse-knowledge online converges with traditional equestrian cultures in ways that can challenge knowledge exchange between veterinarians, farriers and trainers. Using a mixed-methods methodology comprising focus groups and questionnaires, Broms, Boije af Gennäs, Radmann and Hedenborg explore equestrians' online repertoires. The analysis demonstrates participants' dissatisfaction with the quality and availability of horse-knowledge, their critical appraisal of online sources and levels of (dis)trust. The authors conclude that overall better quality online information is desired, but current online repertoires differ between participants based on experience (e.g., those with less horse experience use online sources more) and socio-economic relations of power.

“*Exploring the Notion of Literacy Within Physical Literacy*” provides an important discussion on the historical and contemporary uses of these concepts with a particular focus on physical literacy as the promotion of lifelong physical activity (Durden-Myers et al.). The growing popularity of physical literacy as a framework of both understanding and action certainly necessitates further critical discussion. The authors analyse an impressive range of studies and offer differing perspectives and questions about the concept's etymology, interpretation and application. Using these insights, the authors are able to describe a key tension around the fluidity and ‘in-betweenness' of its interpretation(s) against desires to measure and capture it empirically. They advocate a pluralist, inclusive approach to physical literacy, understanding it as an embodied physical capacity that is central to other forms of literacy and therefore key to our understanding of the world and others around us.

Gender inequalities persist within sport. “*Women's Volunteering and Voluntary Leadership Positions in Sport—Secondary Analyses of the German Survey on Volunteering*” provides a focused contribution to this evidence base, using an intersectional approach (Burrmann and Sielschott). Burrmann and Sielschott base their study on a quantitative population survey on volunteering in Germany with more than 25,000 respondents (2014 and 2019). The findings reveal that the proportion of women who volunteer is lower than that of men; gender differences emerge in motives and impulses for volunteering; and fewer women hold leadership positions. Intersectional inequalities also impact upon volunteering. Volunteering increases relative to higher incomes, A-Level education, no-immigration status, marriage, and age (youth). Similarly, the likelihood of holding leadership positions decreases based on gender, immigration status and having children living within the household. These findings raise important considerations for practice and policy approaches aiming to close the gender gap in sport.

## Author contributions

All authors listed have made a substantial, direct, and intellectual contribution to the work and approved it for publication.

## Conflict of interest

The authors declare that the research was conducted in the absence of any commercial or financial relationships that could be construed as a potential conflict of interest.

## Publisher's note

All claims expressed in this article are solely those of the authors and do not necessarily represent those of their affiliated organizations, or those of the publisher, the editors and the reviewers. Any product that may be evaluated in this article, or claim that may be made by its manufacturer, is not guaranteed or endorsed by the publisher.
